# Investigating the Role of Methylation in Silencing of *VDR* Gene Expression in Normal Cells during Hematopoiesis and in Their Leukemic Counterparts

**DOI:** 10.3390/cells9091991

**Published:** 2020-08-29

**Authors:** Urszula Nowak, Sylwia Janik, Aleksandra Marchwicka, Agnieszka Łaszkiewicz, Agnieszka Jakuszak, Małgorzata Cebrat, Ewa Marcinkowska

**Affiliations:** 1 Laboratory of Protein Biochemistry, Faculty of Biotechnology, University of Wrocław, Joliot-Curie 14a, 50-383 Wrocław, Poland; urszula.nowak.bio@gmail.com (U.N.); aleksandra.marchwicka2@uwr.edu.pl (A.M.); agjakuszak@gmail.com (A.J.); 2 Laboratory of Molecular and Cellular Immunology, Department of Tumor Immunology, Institute of Immunology and Experimental Therapy, Polish Academy of Science, Weigla 12, 53-114 Wrocław, Poland; sw90@interia.pl (S.J.); bijbo@interia.pl (A.Ł.)

**Keywords:** blood cells, vitamin D receptor, gene expression, hematopoietic stem cells, hematopoietic progenitor cells, differentiation, methylation, leukemia

## Abstract

(1) *Background*: Vitamin D receptor (VDR) is present in multiple types of blood cells, and its ligand, 1,25-dihydroxyvitamin D (1,25D), is important for the proper functioning of the immune system. Activity of VDR is higher in hematopoietic stem and progenitor cells than in fully differentiated blood cells of mice and humans. In some human acute myeloid leukemia (AML) blasts, the expression of the *VDR* gene is also high. The mechanism of silencing the *VDR* gene expression during differentiation of blood cells has been addressed in this work. (2) *Methods*: The cells have been obtained using fluorescence activated sorting from murine tissues and from human umbilical cord blood (UCB). Then, the expression of the *VDR* gene and transcriptional activity of the VDR protein has been tested in real-time polymerase chain reaction (PCR). Eventually, the methylation of *VDR* promoter regions was tested using bisulfite sequencing. (3) *Results*: The CpG islands in *VDR* promoters were not methylated in the cells studied both in mice and in humans. The use of hypomethylating agents had no effect toward expression of human *VDR* transcripts, but it increased expression of the VDR-target gene, *CYP24A1*. (4) *Conclusions*: The expression of the *VDR* gene and transcriptional activity of the VDR protein varies at successive stages of hematopoietic differentiation in humans and mice, and in blasts from AML patients. The experiments presented in this case indicate that methylation of the promoter region of the *VDR* gene is not the major mechanism responsible for these differences.

## 1. Introduction

The vitamin D receptor (VDR) belongs to the super-family of nuclear receptors, which function as ligand-activated transcriptional regulators [1]. 1,25-Dihydroxyvitamin D (1,25D) is a natural ligand for VDR, and the 1,25D-VDR complex regulates transcription of approximately 3% of mammalian genes [2]. The most important function of the 1,25D-VDR system is the maintenance of healthy bones by regulating calcium-phosphate homeostasis [3]. However, the VDR binds not only 1,25D, but also a litocholic acid (LCA) [4], which is a toxic and carcinogenic product of bile acids metabolism. Since VDR is ubiquitous in lower level organisms, where there is no need for bone maintenance, the proposed role of VDR in these organisms has been in detoxification [5]. However, in mammals, VDR is widely present in tissues neither related to calcium-phosphate homeostasis nor to detoxification. Therefore, its role is believed to be broader than just bone protection and detoxification. One such role, yet not fully understood, is in modulation of the immune system [6]. All immune cells are produced in the process of hematopoiesis from hematopoietic stem cells (HSCs) through hematopoietic progenitor cells (HPCs) [7]. Therefore, the question whether or not 1,25D-VDR affect certain steps of hematopoiesis has been addressed. It has been documented that VDR is present and transcriptionally active in human and murine hematopoietic stem and progenitor cells (HSPCs) [8]. 1,25D induced HSPCs proliferation in zebrafish and enhanced hematopoietic colony numbers in humans [9]. Another study has shown that, in HSCs exposed to physiological concentrations of 1,25D, markers of monocytic differentiation were induced [10]. In HPCs exposed to 1,25D, the balance between granulocyte/macrophage colony forming units (CFU-GM) and monocyte/macrophage colony forming units (CFU-M) is shifted to favour the later ones [11]. The protein level and transcriptional activity of VDR is higher in HSPCs than in differentiated blood cells, and is higher in monocytes than in other lineages [8,12]. It is, therefore, interesting how the expression of the gene encoding VDR is being silenced during differentiation of certain hematopoietic pathways.

One of the mechanisms of gene silencing during cell differentiation is DNA methylation, which is an epigenetic regulatory process in which cytosine residues are methylated within CpG dinucleotides, in the regions with a high frequency of CpG sites, called CpG islands [13]. The *VDR* gene itself has been reported as regulated by DNA methylation at CpG islands such as an adaptation to the light exposure [14]. Therefore, we studied if the mechanism of *VDR* silencing throughout the process of hematopoiesis is mediated by methylation of *VDR* promoter regions. We decided to study blood differentiation in murine and human models. This was because the structure of murine and human *VDR* genes are different. Human 5′ regulatory region is very complex, and consists of seven untranslated exons (1a–1g) and three promoter regions [15], while, in the murine 5′ UTR region, only two exons (1 and 2) with one promoter were identified [16]. Moreover, the regulation of *VDR* expression is diverse in humans and mice since, in human hematopoietic cells, the expression of *VDR* is up-regulated by all-*trans*-retinoic acid (ATRA), and not by 1,25D, while, in murine hematopoietic cells, the expression of *Vdr* is auto-regulated by 1,25D, but not by ATRA [8]. For this study, blood cells at various steps of hematopoiesis have been isolated using the fluorescence activated cell sorting (FACS) method, and the methylation of CpG islands was tested by DNA sequencing after bisulfite conversion of methylated DNA.

## 2. Materials and Methods

### 2.1. Isolation of Murine Blood Cells

The experiments using animals were performed according to the procedures approved by the First Local Ethical Commission for Animal Experimentation in Wrocław at the Institute of Immunology and Experimental Therapy (permit numbers 21/2016/W, 21/2016/U, 20/2016/U issued on 5 January 2016). Bone marrow cells were isolated from 8-week-old C57BL/6 mice by washing the femur and tibia with ice-cold phosphate-buffered saline (PBS, Biowest, Nuaillé, France) stream. All tissues and cells were dissociated by the syringe trituration, washed twice with PBS by centrifugation (400 rcf, 5 min, 4 °C), and re-suspended in PBS supplemented with 2% fetal bovine serum (FBS, Sigma-Aldrich, St. Louis, MO, USA). Mature T and B lymphocytes were isolated from spleen cells stained with anti-CD3-APC and anti-CD19-PE antibodies (Becton Dickinson, San Jose, CA, USA), respectively. Granulocytes were isolated from the bone marrow stained with anti-CD45-FITC antibody (Becton Dickinson, San Jose, CA, USA), using CD45/SSC-based sorting criteria. Stained cells were then sorted using FACS-Aria sorter (Becton Dickinson, San Jose, CA, USA).

### 2.2. Isolation of Human Hematopoietic Stem and Progenitor Cells

Human umbilical cord blood UCB was obtained by the obstetricians post-delivery from umbilical cords of mothers who gave informed consent for this study. The study was accepted by the local Ethical Committee (permit No KB-394/2015). RosetteSep™ Human Hematopoietic Progenitor Cell Enrichment Cocktail (StemCell Technologies, Cologne, Germany) was used to enrich HSPCs cells. RosetteSep™ Cocktail was added to 15 mL of cord blood and incubated for 20 min at room temperature. After that, cord blood was diluted using PBS supplemented with 2% FBS in a 1:1 ratio. Histopaque 1077 (Sigma-Aldrich, St. Louis, MO, USA) was added to SepMate™ Tube, and then diluted blood was carefully layered onto gradient medium, and centrifuged at 1200× *g* for 20 min. The opaque interface containing mononuclear cells was moved to a fresh sterile tube and washed three times with PBS supplemented with 2% FBS. Then the cells were stained with the following antibodies: CD10-FITC and CD38-APC from Biolegend and CD34-PE from ImmunoTools (Friesoythe, Germany). Stained cells were then sorted out using MoFlo XDP, Cell Sorter (Beckman Coulter, Brea, CA, USA). Hematopoietic cell populations were sorted according to the Becton Dickinson manual [17]. The following populations were obtained: HSCs CD34+, CD10-, CD38- (this population includes also multipotent progenitors), common lymphoid progenitors (CLPs) CD34+, CD10+, CD38-, and common myeloid progenitors (CMPs) CD34+, CD38+, CD10-. The cells were transferred to Stemline^®^ Hematopoietic Stem Cell Expansion Medium (Sigma-Aldrich) containing 4 mM L-glutamine, 100 units/mL penicillin, 100 μg/mL streptomycin, and growth factors (100 ng/mL stem cell factor (SCF), granulocyte colony stimulating factor (G-CSF) and thrombopoietin (TPO), 50 ng/mL FMS-like tyrosine kinase 3 ligand (FLT3L) from ImmunoTools) and maintained at standard cell culture conditions.

### 2.3. Cell Lines and Acute Myeloid Leukemia Cells

HL60 and Jurkat cells were acquired from the local cell bank at the Institute of Immunology and Experimental Therapy in Wrocław (Poland), while KG1 cells were purchased from the German Resource Center for Biological Material (DSMZ GmbH, Braunschweig, Germany). The cells were cultured in RPMI-1640 medium (Biowest, Nuaillé, France) with 10% FBS, 2 mM L-glutamine, 100 units/mL penicillin, and 100 μg/mL streptomycin (all from Sigma-Aldrich) and maintained at standard cell culture conditions. DNA from AML patients’ blasts were kept frozen at −80 °C from the study published before [18].

In order to study the influence of hypomethylating agents, HL60 cells were exposed to 10 nM 1,25D, 1 μM ATRA, 50 or 100 μM zebularine (ZEB), and/or 5 or 10 μM 5-azacytidine (5AZA) for 96 h. The expression of cell surface markers of differentiation was determined by flow cytometry. Cells were washed and stained with antibodies: CD11b-FITC and CD14-PE (ImmunoTools) for 1 h on ice. Next, they were washed with ice-cold PBS and suspended in 0.5 mL of PBS supplemented with 0.1% bovine serum albumin (BSA) prior to analysis on Accuri C6 (Becton–Dickinson). Data analysis was performed using FCS Express (De Novo™ Software, Pasadena, CA, USA).

### 2.4. cDNA Synthesis and Real-Time Polymerase Chain Reaction (PCR)

For PCR analyses, the cells were stimulated with 10 nM 1,25D and/or 1 μM ATRA for 96 h. RNA from unstimulated and stimulated cells was isolated using either RNA Purification Columns or PicoPure RNA Isolation Kit (ThermoFisher Scientific, Waltham, MA, USA), according to manufacturer’s recommendations. Reverse transcription was done with iScript SuperMix (BioRad) or High-Capacity cDNA Reverse Transcription Kit (ThermoFisher Scientific) using random hexamers. The real-time PCR analysis was performed using Real-Time PCR–PowerUp™ SYBR Green Master Mix (Applied Biosystems, Foster City, CA, USA) on BioRad CFX Connect apparatus (Bio-Rad Laboratories Inc., Hercules, CA, USA) or SensiFAST SYBR^®^ No-ROX Kit (Bioline, London, UK) using CFX Real-Time PCR System (Bio-Rad). For murine samples, the reaction consisted of 40 cycles (95 °C for 15 s and 60 °C for 60 s). The thermal profile for human samples consisted of 45 cycles (95 °C for 5 s, 54/58 °C for 10 s, 72 °C for 5 s.

The following primers were used:

murine *Gapdh* forward 5′AACTTTGGCATTGTGGAAGG3′, reverse 5′ ACACATTGGGGGTAGGAACA3′;
murine *Vdr* forward 5′CACCTGGCTGATCTTGTCAGT 3′, reverse 5′ CTGGTCATCAGAGGTGAGGTC 3′;
murine *Cyp24a1* forward 5′CACGGTAGGCTGCTGAGATT 3′, reverse 5′ CCAGTCTTCGCAGTTGTCC 3′;
human *GAPDH* forward 5′-CATGAGAAGTATGACAACAGCCT-30, reverse 5′-AGTCCTTCCACGATACCAAAGT-3′;
human *VDR* forward 5′-CCTTCACCATGGACGACATG-30, reverse 5′-CGGCTTTGGTCACGTCACT-3′;
human *VDR1C* forward 5′-GGGTCTGAAGTGTCTGTGAGA-3′, reverse 5′-GAAGTGCTGGCC GCCATTG-3′;
human *CYP24A1* forward 5′-CTCATGCTAAATACCCAGGTG-30, reverse 5′-TCGCTGGCAAAACGCGATGGG-3′;

Relative quantification of gene expression was analyzed with the ΔΔCt method using either glyceraldehyde 3-phosphate dehydrogenase gene (*Gapdh*) or *GAPDH* as the endogenous controls [19].

### 2.5. Analysis of Methylation in Promoter Regions of Murine and Human VDR

CpG islands were identified using MethPrimer software [20] and the following criteria were used: island size > 100, GC Percent > 50.0, Obs/Exp > 0.6. For murine samples, the bisulfite conversion was performed as previously described [21]. In addition, 5 µL of the converted DNA was taken for the outer reaction and 2.5 µL of the outer reaction products was taken for the inner reaction. The cycling conditions for both (outer and inner) 25 µL reactions consisted of 35 cycles (95 °C for 30 s, 50 °C for 30 s, and 72 °C for 30 s). For human samples, DNA was isolated using DNA Extractme Genomic DNA Kit or Extractme DNA Blood Kit (Blirt, Gdańsk, Poland), according to the manufacturer’s recommendations. Next, EZ DNA Methylation™ Kit (Zymo Research, Irvine, CA, USA) was used to perform the bisulfite conversion of DNA. The nested PCR analysis was performed using DreamTaq™ PCR Master Mix (Thermo Fisher Scientific). The cycling conditions for both (outer and inner) 50 µL reactions consisted of 35 cycles (95 °C for 30 s, 56.5/60/62 °C for 30 s, and 72 °C for 60 s).

The following primers were used:

murine *Vdr* forward outer and inner: 5′-GAGAAATTTATTTGAGGTTTTTTATT-3′;
murine *Vdr* reverse outer: 5′-CCAACCACAATACAACACAAAC-3′;
murine *Vdr* reverse inner: 5′-TAATTCTACCCAATCTACTATAAAC-3′;
human *VDR1A* forward outer 5′-GTTGGGTTGTTTTTGTTTGTTAAA -3′, reverse outer 5′-TCAAACCTCAATACCCCTTAATATC-3′;
human *VDR1C* forward outer 5′-GGGATTAAAGTTTTTGGAAAGAGTT-3′, reverse outer 5′-CACCTACCTAAAAAAACAAAAAACAA-3′;
human *VDR1A_1* forward inner 5′-TGGGTTGTTTTTGTTTGTTAAAAG-3′, reverse inner 5′-CCCTATCCTAAAACCCCCTTTC-3′;
human *VDR1A_2* forward inner 5′-GAAAGGGGGTTTTAGGATAGGG-3′, reverse inner 5′-TACCCC TTAATATCCCAACCTC-3′;
human *VDR1C* forward inner 5′-GTTTATTTTTTTAGAGATTGGGG-3′, reverse inner 5′-ATCTCACAAACACTTCAAAC-3′;

PCR products were resolved on 3% or 2% agarose gels and purified using Monarch^®^ PCR and DNA Cleanup Kit (New England Biolabs, Ipswich, MA, USA). The amplification products were cloned into pGEM^®^-T Easy Vector (Promega Corporation, Madison, WI, USA), transformed into E. coli HST08 strain and subjected to blue/white selection. Plasmids from several white colonies were then purified and sequenced using T7 (5′-TAATACGACTCACTATAGG-3′) or SP6 (5′-ATTTAGGTGACACTATAG -3′) primers.

### 2.6. Statistical Analysis

The sample distribution was assessed using the Shapiro-Wilk test. For samples with normal distribution, the *t*-test was used to assess a significance of differences. For the remaining samples, a non-parametric one-way ANOVA test followed by a Mann-Whitney U-test was used for assessing the significance of the differences between samples.

## 3. Results

### 3.1. Lack of Methylation in the Promoter Region of the Murine Vdr Gene in Murine Blood Cells

**Figure 1:**
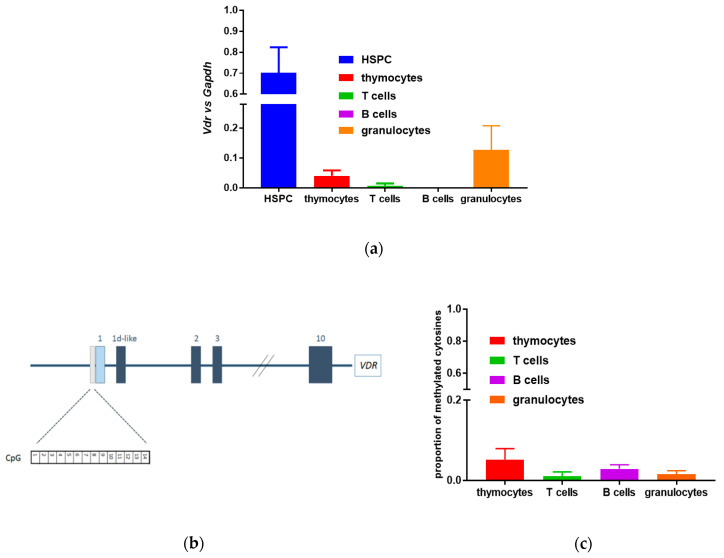
Expression of *Vdr* in different blood cells and methylation of CpG island in the promoter region of murine *Vdr*. (**a**) mRNA was isolated from murine blood cells and *Vdr* expression was measured by real-time PCR relative to *Gapdh* mRNA levels. Expression in kidney cells was used as a calibrator. The bars represent mean values (±SEM) of the fold changes in mRNA levels. (**b**) A CpG island was identified in the promoter region of murine *Vdr* gene using MethPrimer software. The localization of the CpG island relative to *Vdr* exons is presented. (**c**) The methylation pattern of the CpG island was examined in thymocytes, splenic mature T and B lymphocytes, and granulocytes from the bone marrow from 11 to 18 mice. The mean (± standard error of the mean (SEM)) proportion of methylated cytosines in the island is presented on the graph.

Since the transcriptional activity of VDR protein was significantly higher in murine HSPCs than in fully differentiated blood cells [8], we hypothesized that epigenetic silencing of *Vdr* gene expression may underlie the observed effect. We have identified a CpG island in the promoter region, localized at −470 to −210 nucleotides relative to the transcriptional start site of the murine transcript. The island has 62% GC content, CpG Obs/Exp = 0.88, and contains 22 CpG dinucleotides. We have analyzed the methylation status 14 CpG dinucleotides contained in a fragment (−445 to −304) of this region using bisulfite conversion (Figure 1a). The following cells were used: thymocytes, splenic mature T and B lymphocytes, and granulocytes from the bone marrow. We have found that, regardless of the VDR transcriptional activity in a given cell type, the methylation of the CpG island associated with the *Vdr* promoter was low, which ranged from 1.5% to 6% (Figure 1b and Figure S1). We, therefore, conclude that CpG island methylation is not responsible for down-regulating of *Vdr* expression in the differentiated blood cells.

### 3.2. Expression of VDR Gene and Activity of VDR Protein in Human Hematopoietic Stem and Progenitor Cells

**Figure 2:**
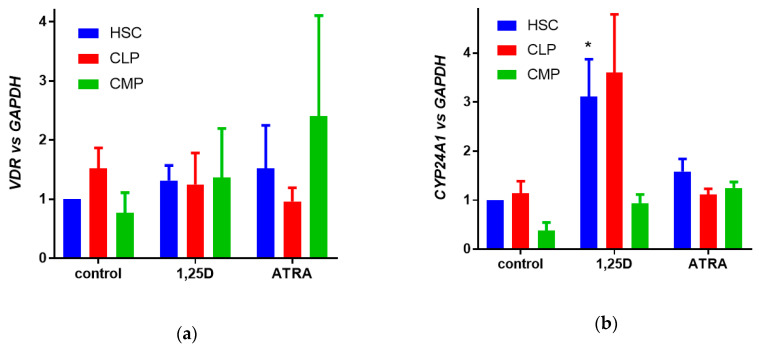
Gene expression in human hematopoietic cells. HSCs, CLPs, and CMPs were sorted from human UCB and then exposed for 96 h to either 10 nM 1,25D or 1 µM ATRA. The expression of *VDR* (**a**) or *CYP24A1* (**b**) was measured by real-time PCR. The bars represent mean values (±SEM) of the fold changes in mRNA levels relative to *GAPDH* mRNA levels obtained in three experiments. Expression in control HSCs (exposed to vehicle) was treated as a calibrator. Value significantly different from this obtained from respective control cells is marked with an asterisk (* *p* < 0.05).

In the next step, we studied the expression of the gene and activity of the VDR protein in human cells at various stages of hematopoiesis. Hematopoietic progenitor cells were isolated from the UCB using Hematopoietic Progenitor Cell Enrichment Cocktail, and in further steps, HSCs, CLPs, and CMPs were isolated using fluorescence activated cell sorting. These cells were either exposed to 10 nM 1,25D, or to 1 µM ATRA or to equivalent volume of solvent for 96 h, and then the relative expression of *VDR* (Figure 2a) and *CYP24A 1* (Figure 2b) genes was studied in real-time PCR using *GAPDH* as a reference gene. In human cells, transcriptional activity of the VDR protein was the highest in HSCs, as presented in Figure 2b, where the significant upregulation of *CYP24A1* expression was documented.

### 3.3. Methylation in Promoter Regions of Human VDR Gene in Human Blood Cells

**Figure 3:**
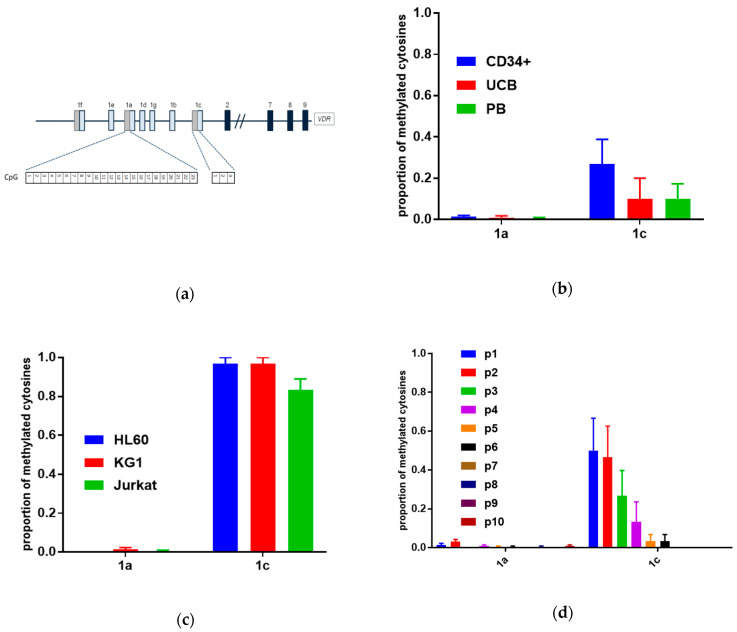
Methylation of CpG islands in the promoter regions of human *VDR*. (**a**) CpG islands were identified in the promoter regions of human *VDR* gene using MethPrimer software. The localization of the CpG islands relative to *VDR* exons is presented. Methylation patterns of CpG islands were examined in (**b**) normal human blood cells, (**c**) human leukemic cell lines, and (**d**) human AML blasts. The mean (± SEM) proportion of methylated cytosines in the respective CpG islands are presented on the graphs.

In the next step, the CpG islands have been identified in human *VDR* gene promoter regions. We have identified a CpG island within the promoter region associated with exon 1a localized at −115 to +216 nucleotides relative to the transcriptional start site of the transcript. The island has 71% GC content, CpG Obs/Exp = 0.84, and contains 32 CpG dinucleotides. We have analyzed the methylation status 23 CpG dinucleotides within a fragment of this region (−10 to +249). Additionally, we have analyzed the methylation status of 3 CpG dinucleotides present (−74 to +40 relative to TSS) in the promoter associated with exon 1c. Methylation of cytosines within this region has been previously reported in T cells from patients with multiple sclerosis [22], even though this region did not fulfill the criteria of a CpG island (Figure 3a). Methylation patterns of the *VDR* gene promoter regions were examined in normal human HSPCs (CD34-positive cells), mononuclear cells from UCB, and in mononuclear cells from peripheral blood (PB) (Figure 3b), leukemic cell lines (Figure 3c), and AML patients (Figure 3d) using bisulfite sequencing. The characteristics of AML patients is presented in Table 1.

Our experiments have shown that the CpG island in the promoter region of exon 1a in all samples was unmethylated. The promoter region of exon 1c was fully methylated in leukemic cell lines. In HSPCs, it was methylated in almost 50%. In UCB and in PB, it was methylated in 10–20%. The CpG dinucleotides in the 1c promoter were not methylated in patients p7–p10, were methylated below 50% in patients p3–p6, while, in patients p1 and p2, they were methylated in around 50%. Detailed results are presented in Figure S2.

### 3.4. Influence of Hypomethylating Agents on 1,25D-Induced Differentiation of HL60 Cells

**Figure 4:**
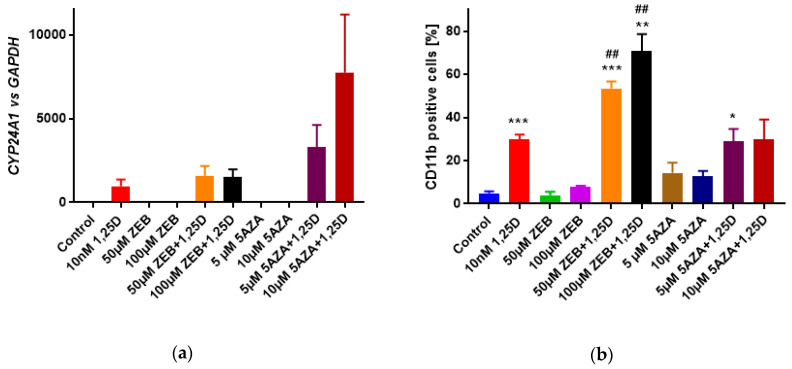
Effects of hypomethylating agents towards 1,25D-induced differentiation of HL60 cells. The cells were exposed to 10 nM 1,25D ± ZEB (50 or 100 µM) or 5AZA (5 or 10 µM) for 96 h. (**a**) Then the expression of *CYP24A1* relative to *GAPDH* mRNA levels was measured in three experiments. The bar charts show mean values (±SEM) of relative quantity. (**b**) The levels of the CD11b cell surface marker were tested in flow cytometry. The bar charts show mean values (±SEM) of percentages of positive cells obtained in three experiments. Values that differ significantly from those obtained for control cells are marked with asterisks (* - 0.05, ** - 0.01, *** - 0.001) while the values that differ significantly from those obtained for cells exposed to 1,25D are marked by hash tags (## - 0.001).

Since the CpG region in the promoter of exon 1c in leukemia cell lines was fully methylated, we hypothesized that hypomethylating agents, zebularine (ZEB) or 5-azacytidine (5AZA), may increase *VDR* expression, and, in consequence, the activity of VDR as a transcription factor. Therefore, exposing HL60 cells to VDR ligand, 1,25D together with hypomethylating agents should increase 1,25D-induced gene expression and cell differentiation. The mRNA was isolated and transcribed to cDNA, and the relative expression of *VDR*, *VDR1C*, and *CYP24A1* genes was studied in real-time PCR using *GAPDH* as a reference gene. The expression of *VDR* and *VDR1C* was at a comparable level in all samples studied (not shown), while the expression of *CYP24A1* was higher in the cells exposed to 1,25D + 10 µM 5AZA than in the cells exposed to 1,25D alone, but the difference was not statistically significant (Figure 4a). Then HL60 cells were exposed to 1,25D ± ZEB or 5AZA for 96 h. Afterward, the differentiating effects of 1,25D or/and hypomethylating agents were examined in flow cytometry. Figure 4b shows that the level of differentiation marker CD11b was significantly increased in samples exposed to 1,25D + ZEB when compared to 1,25D alone. The cell surface marker CD14 was at comparable levels in samples exposed to 1,25D alone and 1,25D + hypomethylating agents (Figure S3). However, it should be noted that, while ZEB did not inhibit HL60 cells viability, 5AZA has inhibited it significantly (Figure S4).

Since, as mentioned above, the expression of *VDR* and *VDR1C* has not been changed by exposure of the HL60 cells to hypomethylating agents, we decided to verify whether these agents have reduced the methylation status of the *VDR 1c* promoter region. Therefore, we have analyzed the methylation of CpG dinucleotides present in the promoter associated with exon 1c in cells exposed to either ZEB or 5AZA. The results, which are presented in Figure S5 indicate that hypomethylating agents have failed to prevent methylation of these cytosines. Since the concentrations of ZEB and 5AZA used in the above experiments have been very high, and 5AZA was already toxic to the cells, we decided not to increase them anymore.

## 4. Discussion

The importance of vitamin D for the proper function of the immune system is more appreciated. People who are vitamin D deficient, or who have inactivating mutations in VDR do not respond adequately to infections [24]. Since the active form of vitamin D in the organisms is 1,25D, which acts as a ligand to nuclear VDR, the status of this receptor in immune cells is worthy of investigation. In our previous paper, we have documented that the transcriptional activity of VDR is higher at early steps of hematopoiesis than in fully differentiated cells both in mice and in humans [8]. Moreover, the high expression of *VDR* has been documented in many AML cells, which, due to various mutations, have been inhibited in their differentiation pathways [25,26]. Thus, it has been proposed many years ago that the analogs of 1,25D might be used as differentiation-inducing drugs for AML [27]. However, due to high variability between underlying causes of AML, and also due to different levels of VDR in these cells [28], the vitamin D analogs have never been approved as a therapy for this disease [29]. AML is a very heterogeneous group of leukemia in which more than 200 different chromosome translocations and other mutations have been detected [30]. Despite the fact that neither of these mutations affects the *VDR* gene, its expression is variable in different blasts [28].

In contrast to single CpG dinucleotides dispersed throughout the genome, CpG islands are associated with the promoters and usually remain unmethylated, which is a hallmark of transcriptionally active chromatin. The methylation of CpG dinucleotides is associated with gene silencing. However, its temporal and causative relationship with other epigenetic mechanisms, such as histone modification, may vary depending on a particular gene or promoter. Since the CpG methylation pattern is inherited throughout cell generations, this mechanism is a very efficient way to stably silence genes during lineage commitment. Taking into account that the major *VDR* promoter is associated with an evolutionarily conserved CpG island, its methylation could have been a plausible explanation for the observed difference in *VDR* expression throughout hematopoiesis. On the other hand, it has been shown that human *VDR* CpG island becomes methylated in breast cancer and treatment with demethylating agents restored the *VDR* expression and susceptibility to differentiation therapy using 1,25D [31].

The problem of the silencing of *VDR* gene expression has been addressed by the others with regard to blood cells. This year, a paper was published, which reported that *VDR* expression is partially regulated by methylation, and that combined hypomethylating drugs and VDR agonists synergistically induced AML cells’ differentiation [32]. In this paper, the authors have presented their retrospective analysis of the AML cells from the GEO: GSE18700 study available in GenBank, which indicated that the promoter region of *VDR* in these cells was methylated. Moreover, the authors have shown, using eight freshly isolated AML blasts, that 5AZA enhanced differentiation of these blasts induced by a 1,25D analog. On the other hand, the study in which transcriptome profiling and analysis of chromatin accessibility was performed using single cells from normal and leukemic bone marrow, did not reveal any differences in *VDR* gene accessibility at various steps of hematopoiesis. That would suggest that, in the cells used for this study, there were no differences in VDR promoter methylation [33]. That finding was in accordance with our earlier study in which we did not observe significant differences in chromatin accessibility between AML cell lines with either high or low VDR expression [26].

Analysis of the AML blasts in this study revealed that the CpG island in *VDR* major promoter region (1a) was not methylated, and the CpG region in promoter of exon 1c was variably methylated in the analyzed blasts. We did not see any correlation of the methylation status with either the AML subtype or cytogenetic features of these blasts. In addition, in the leukemic cells lines, the major CpG island of the *VDR* gene was not methylated, but the CpG region in the promoter of 1c was fully methylated. Surprisingly, either ZEB or 5AZA at very high concentrations did not stop this region from methylation. The methylation of this region has been shown in T cells of patients with multiple sclerosis, but methylation inhibitors have neither been tested in these patients nor in their cells ex vivo [22]. Therefore, the possibility of changing the methylation status of 1c promoter region in blood cells have not been confirmed. However, we have observed increased expression of a CD11b differentiation marker in the HL60 cell line exposed to ZEB. We suppose that this effect has not been mediated by VDR, but by some other transcription factor necessary for expression of the gene encoding CD11b. We have also observed some increase in *CYP24A1* expression in the cells exposed to 1,25D and 5AZA when compared to 1,25D alone. According to the data from the literature, we hypothesize that this might be due to demethylation in the promoter region of *CYP24A1* [34]. Therefore, we propose that, in human blood cells, both normal and leukemic methylation of the 1c exon promoter does not have functional consequences.

## 5. Conclusions

From this and from our earlier research, we have known that the expression of *VDR* gene and transcriptional activity of the VDR protein varies at different steps of hematopoiesis in humans and mice, and in blasts from AML patients. Our experiments presented in this case indicate that methylation of the promoter region of the *VDR* gene is not the major mechanism responsible for these differences.

## Figures and Tables

**Table 1:** Characteristics of the patients whose acute myeloid leukemia (AML) blasts were studied.

Patient	Age	Sex	Type ^1^	Prognosis Based on Cytogenetics	Methylation of 1c Promoter ^2^
p1	54	M	M5b	intermediate	+++
p2	60	F	M1	poor	+++
p3	79	M	M5b	poor	++
p4	42	M	M2	good	++
p5	54	F	M2	good	+
p6	50	F	M2	intermediate	+
p7	75	F	M1	poor	-
p8	23	M	M2	good	-
p9	79	M	M1	poor	-
p10	74	F	M5b	intermediate	-

^1^ Types of AML according to French-American-British (FAB) classification [23]. ^2^ Based on the results presented in Figure 3d.

## Supplementary Materials

The following are available online at https://www.mdpi.com/2073-4409/9/9/1991/s1. Figure S1: Methylation pattern of *Vdr* gene promoter in murine blood cells. Figure S2: Methylation pattern of *VDR* gene promoter regions in human normal blood and leukemic cells. Figure S3: Effects of hypomethylating agents towards 1,25D-induced CD14 expression on HL60 cells. Figure S4: Effects of hypomethylating agents to viability of HL60 cells. Figure S5: Methylation pattern of *VDR* gene promoter 1c region in HL60 cells exposed to hypomethylating agents.
