# Supplementary material for: Investigating the Role of Methylation in Silencing of VDR Gene Expression in Normal Cells during Hematopoiesis and in Their Leukemic Counterparts
Source: Cells. 2020 Aug 29;9(9):1991. doi: 10.3390/cells9091991 (PMC7563846; doi:10.3390/cells9091991)
Supplement: Supplementary file 1 [file cells-09-01991-s001.pdf]

Figure S1: Methylation pattern of *Vdr* gene promoter in murine blood cells.

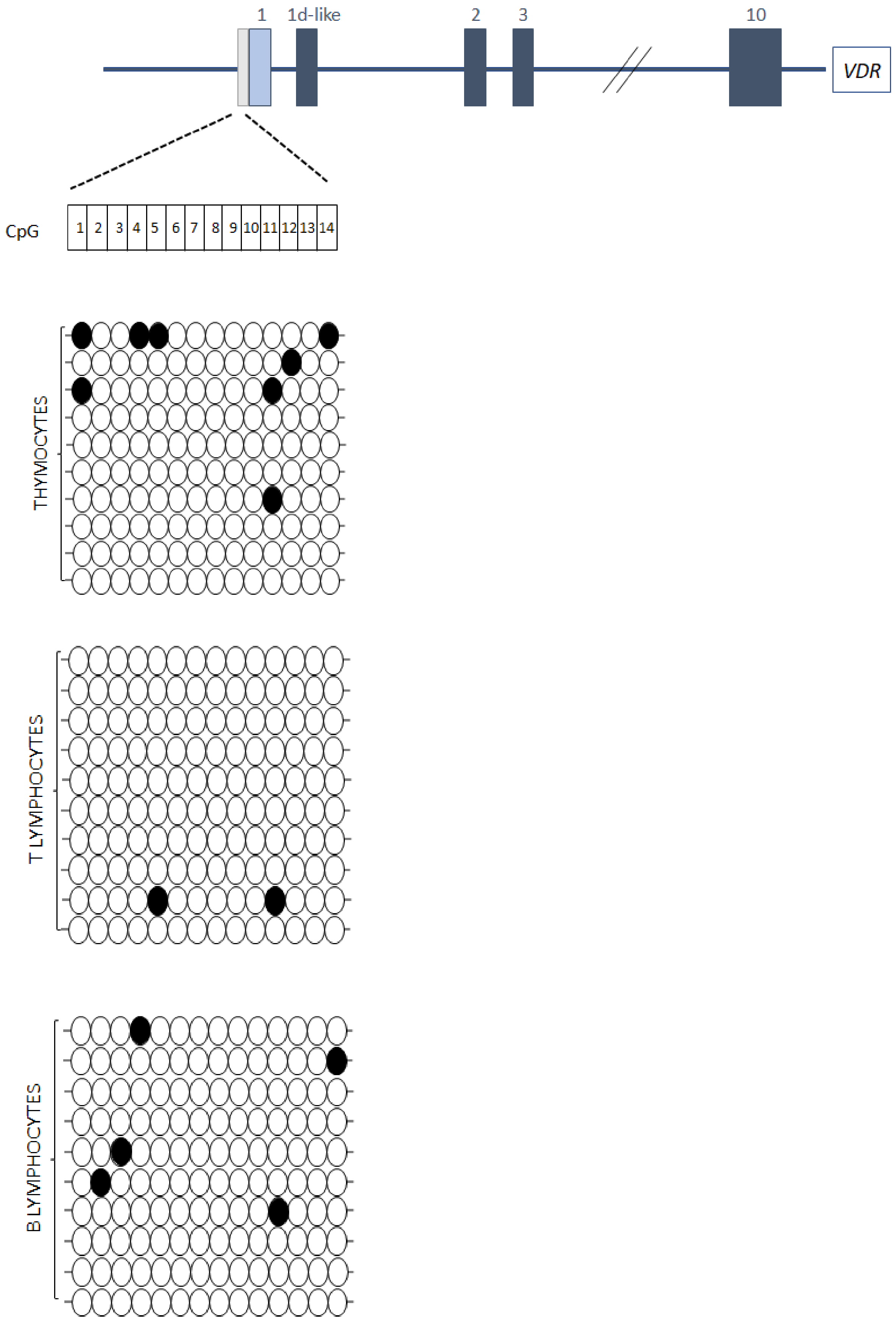

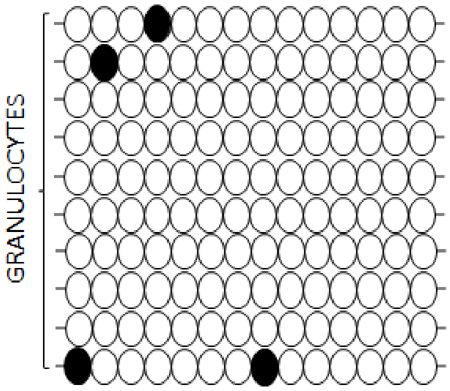

The methylation pattern of 14 CpG dinucleotides in the promoter region of murine *Vdr* was examined in thymocytes, splenic mature T and B lymphocytes, and granulocytes from the bone marrow from 11 to 18 mice. Methylated cytosines are marked by black circles.

Figure S2: Methylation pattern of *VDR* gene promoter regions in human normal blood and leukemic cells.

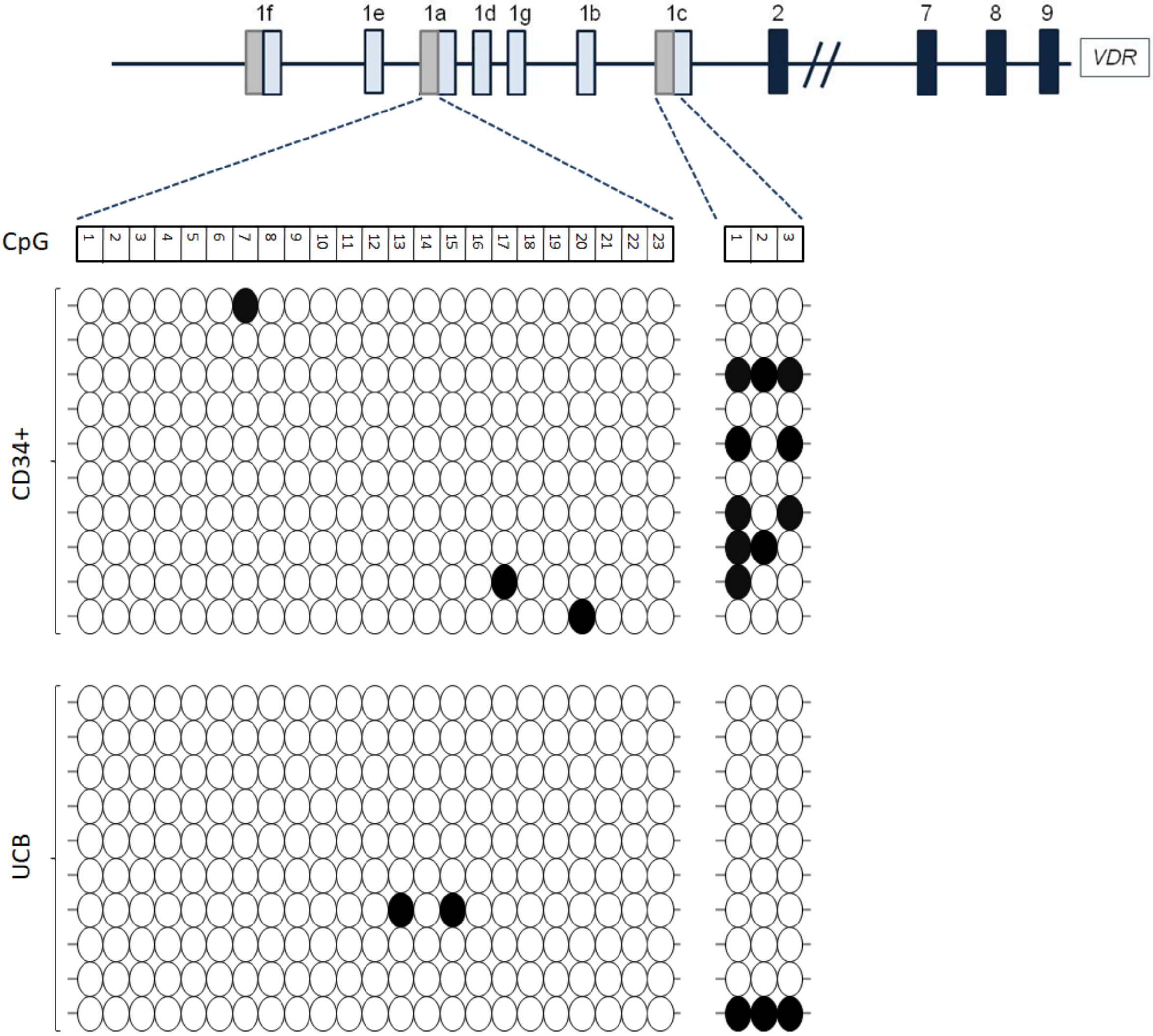

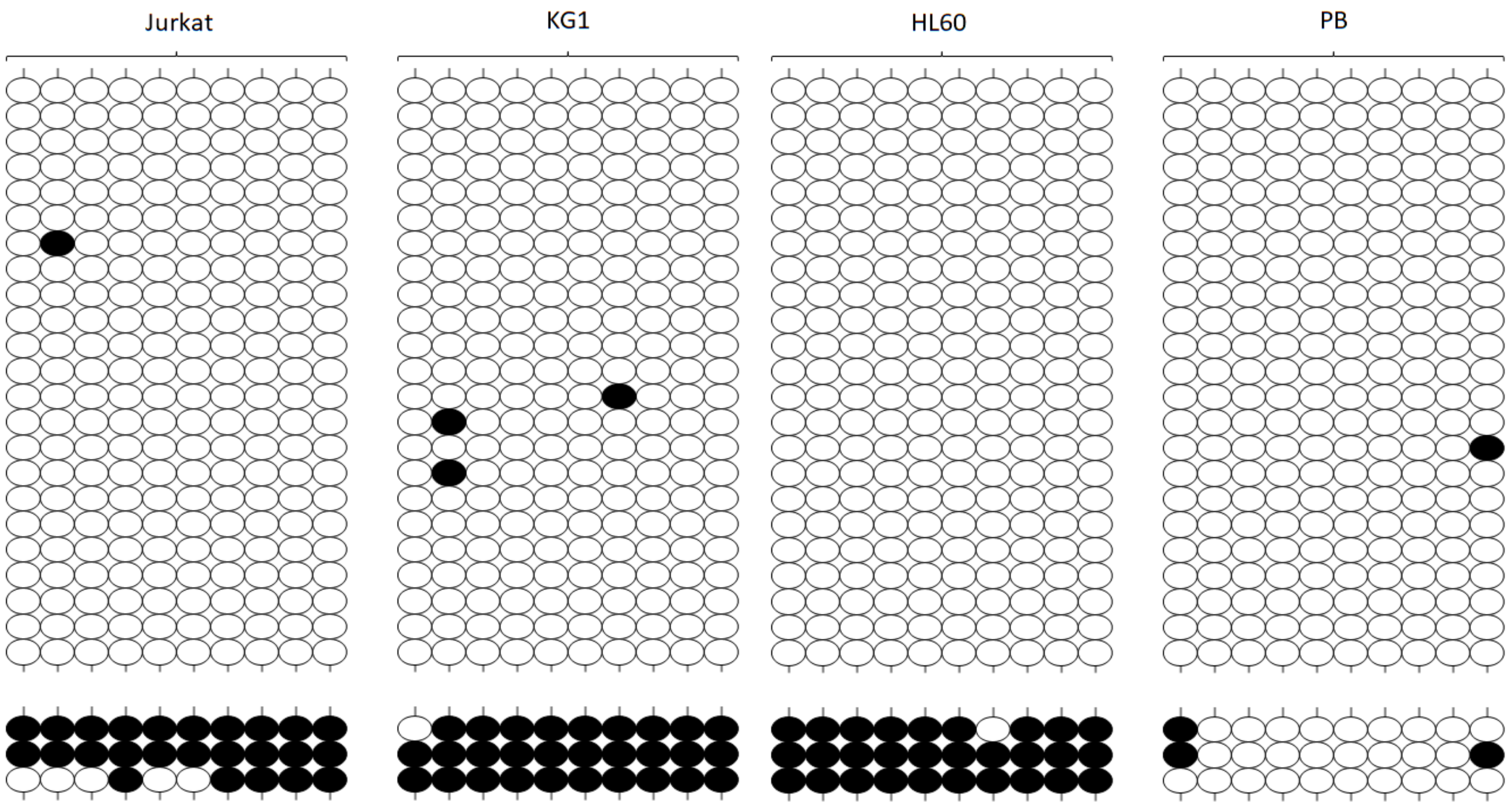
Jurkat
KG1
HL60
PB

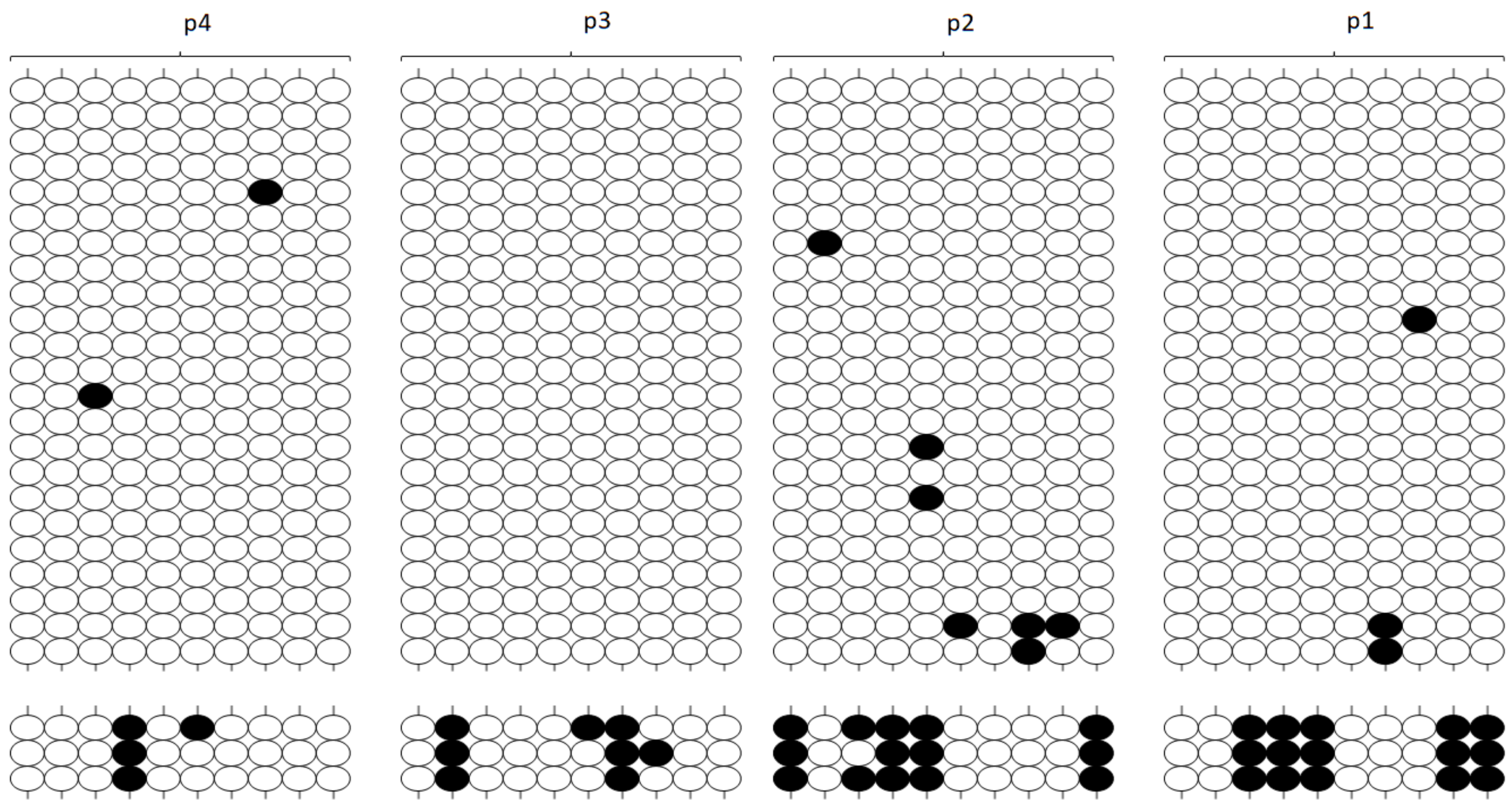
p4
p3
p2
p1

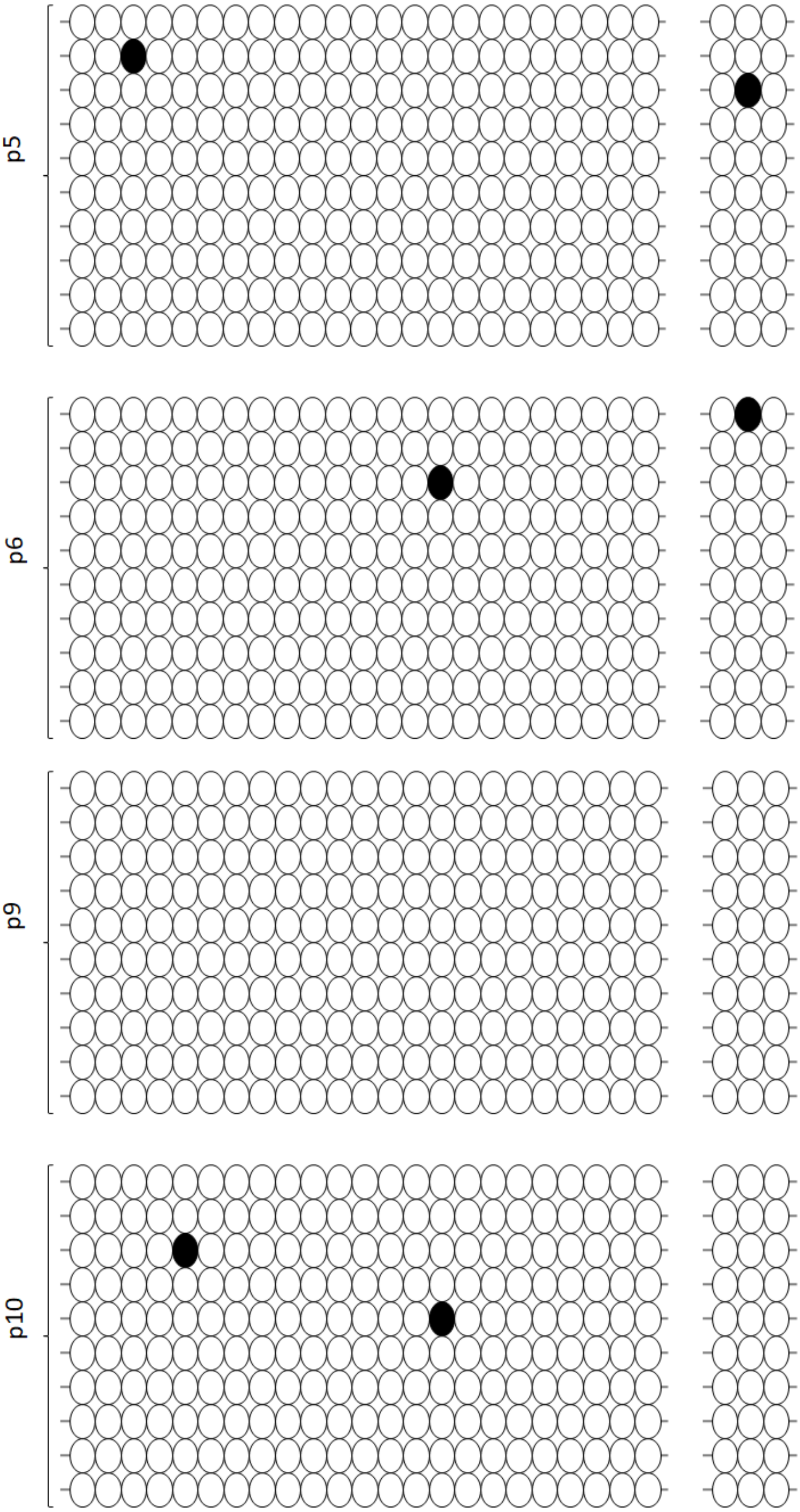

Methylation of CpG islands in the promoter regions of human *VDR*. Methylation patterns of CpG islands were examined in normal human blood cells, human leukemic cell lines, and human AML blasts. Methylated cytosines are marked by black circles.

Figure S3: Effects of hypomethylating agents towards 1,25D-induced CD14 expression on HL60 cells.

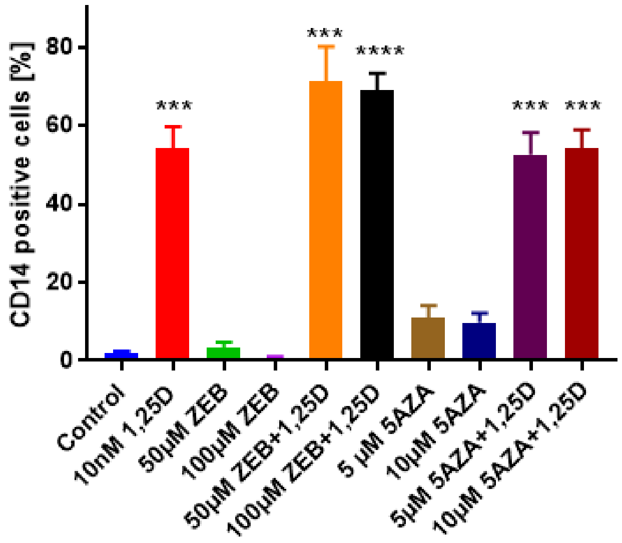

The cells were exposed to 10 nM 1,25D ± ZEB (50 or 100 μM) or 5AZA (5 or 10 μM) for 96 h. The levels of the CD14 cell surface marker were tested in flow cytometry. The bar charts show mean values (±SEM) of percentages of positive cells obtained in three experiments. Values that differ significantly from those obtained for control cells are marked with asterisks (*** - 0,001, **** - 0,0001).

Figure S4: Effects of hypomethylating agents to viability of HL60 cells.

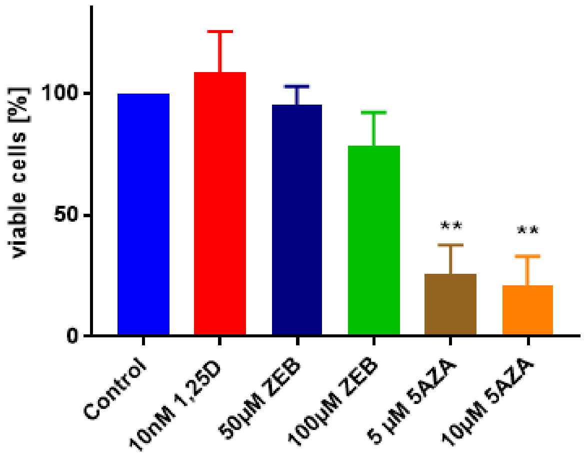

The cells were exposed to ZEB (50 or 100 μM) or 5AZA (5 or 10 μM) for 96 h. Then the viability of the cells was tested using MTT assay. The bar charts show mean values (±SEM) of percentages of viable cells obtained in three experiments. Values that differ significantly from those obtained for control cells are marked with asterisks (** - 0,01).

Figure S5: Methylation pattern of *VDR* gene promoter 1c region in HL60 cells exposed to hypomethylating agents.

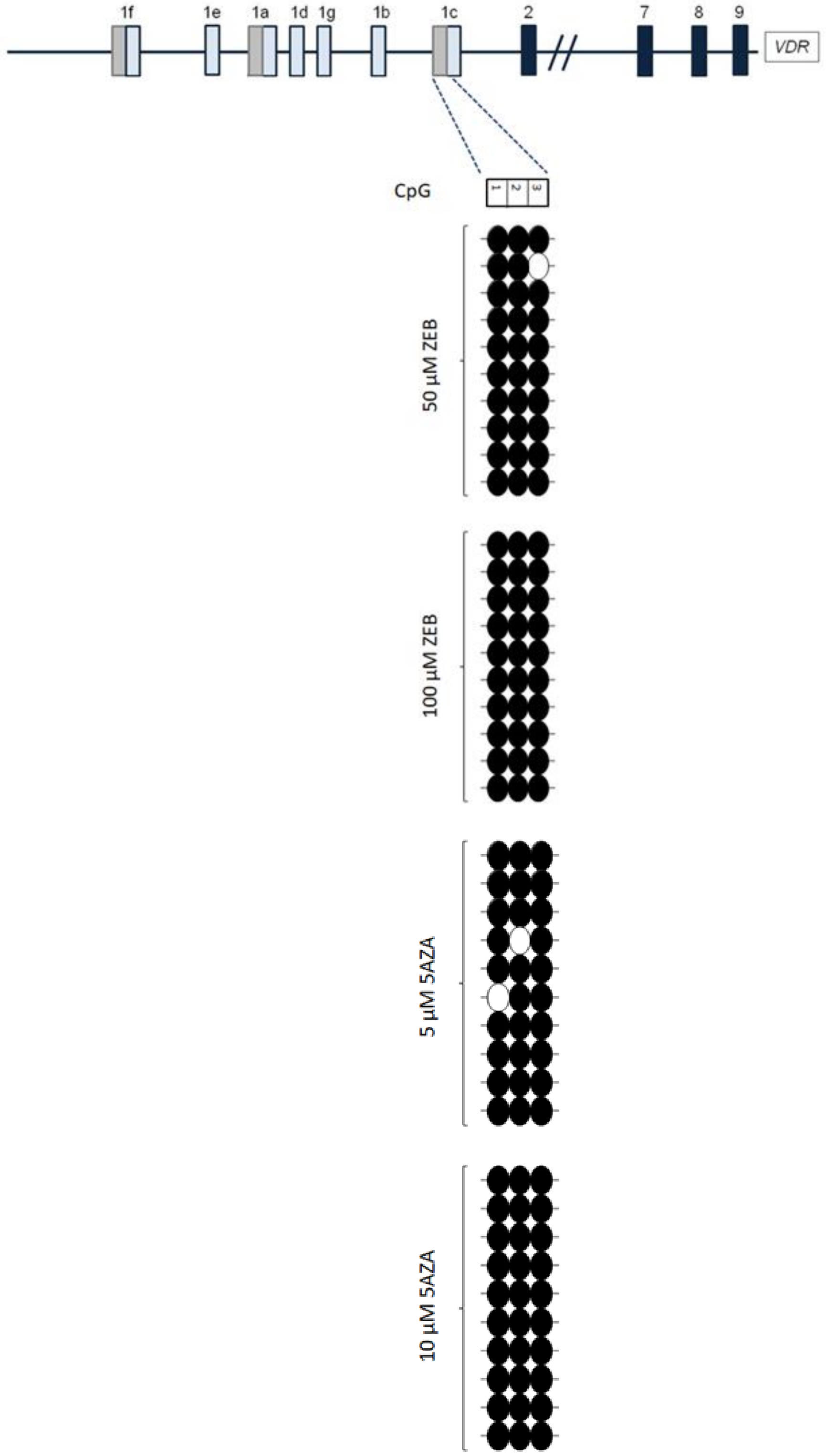

Methylation patterns of CpG dinucleotides were examined in HL60 cells exposed to ZEB (50 or 100 µM) or 5AZA (5 or 10 µM) for 96 h. Methylated cytosines are marked by black circles.
